# *Aphanothece* sp. as promising biostimulant to alleviate heavy metals stress in *Solanum lycopersicum* L. by enhancing physiological, biochemical, and metabolic responses

**DOI:** 10.1038/s41598-023-32870-4

**Published:** 2023-04-27

**Authors:** Soufiane Fal, Abderrahim Aasfar, Ali Ouhssain, Hasnae Choukri, Abelaziz Smouni, Hicham El Arroussi

**Affiliations:** 1 Algal Biotechnology Laboratory, Rabat Design Center, Moroccan Foundation for Advanced Science, Innovation and Research (MASCIR), Rue Mohamed Al Jazouli – Madinat Al Irfane, Rabat, Morocco; 2 Plant Physiology and Biotechnology Team, Center of Plant and Microbial Biotechnology, Biodiversity and Environment, Faculty of Sciences, Mohammed V University in Rabat, Rabat, Morocco; 3 International Center for Agricultural Research in the Dry Areas (ICARDA), Rabat, Morocco; 4 Agrobiosciences Program, University Mohamed 6 Polytechnic (UM6P), Ben Guerir, Morocco

**Keywords:** Biochemistry, Biotechnology, Plant sciences

## Abstract

Heavy metals (H.M) are a major environmental concern around the world. They have harmful impact on plant productivity and pose a serious risk to humans and animals health. In the present study, we investigated the effect of *Aphanothece* crude extract (ACE) on physiological, biochemical, and metabolic responses of tomato plant exposed to 2 mM Pb and Cd. The results showed a significant reduction of tomato plant weights and perturbation in nutrients absorption under 2 mM Pb and Cd conditions. Moreover, ACE treatment showed a significant enhancement of plant biomass compared to plants under Pb and Cd. On the other hand, ACE application favoured H.M accumulation in root and inhibited their translocation to shoot. In addition, ACE treatment significantly enhanced several stress responses in plant under Pb and Cd stress such as scavenging enzymes and molecules: POD, CAT, SOD, proline, and polyphenols etc. Furthermore, ACE treatment showed remodulation of metabolic pathways related to plant tolerance such as wax construction mechanism, particularly SFA, UFA, VLFA, alkanes, alkenes, and sterols biosynthesis to enhance tolerance and resistance to H.M stress. In the present study, we emphasized that ACE alleviates H.M stress by minimizing metal translocation to above-part of plant and enhancing plant growth, nutrients absorption, and biochemical responses.

## Introduction

Agriculture soil contamination with heavy metals (H.M) has been widely reported^1–4^. It can be due to the excessive use of fertilizers, pesticides and sewage irrigation^2,5^. In small quantities, some metals are required to maintain plant growth and development. However, some others like lead (Pb) and cadmium (Cd) even at low level are highly toxic^6^. Plants exposed to these metals exhibited significant changes on physiological, biochemical, and metabolic parameters^7^. Among H.M, Pb and Cd are the most toxic causing a high level of damage to plants growth and food health^3,4^. Pb and Cd can reduce plants growth, preventing photosynthesis, organelles deterioration, generate reactive oxygen species (ROS) which activate antioxidative system and perturbate nutrient uptake and translocation^6–8^.

Different agricultural practices and biotechnology methods were used to restore harmful damages of H.M in plants and improve their resilience to H.M stress^9^. The treatment by exogenous nutriments like Calcium (Ca), Zinc (Zn), Potassium (K) etc., phytohormones, exogenous molecules like Hydrogen peroxide (H_2_O_2_), and some plant biostimulants like selenium and plant-growth promoting rhizobacteria (PGPR), were reported^10–12^. Furthermore, microalgae (eukaryotic) and cyanobacteria (procaryotic) are potentially diverse, ubiquitous and dominant group of photosynthetic organisms in nature^13,14^. Microalgae were used in different application fields including phycoremediation. In this regard, numerous studies showed the robustness of microalgae in wastewater treatment and H.M removal using their antioxidant system and accumulation of some metabolites like polyunsaturated fatty acids (PUFA), Alkanes, Sterols etc., for H.M stress resistance and tolerance^15,16^.

Recently the use of microalgae and cyanobacteria as promising biostimulant of plant growth, performance and resistance to abiotic and abiotic stress was investigated in numerous studies^11,17–19^. Moreover, *Aphanothece* sp. extract as a biostimulant showed improvement of *Solanum lycopersicum* growth, nutrients uptake, antioxidative system and tolerance to abiotic and biotic stress e.g. salinity^18–20^. Thus, we picked attention for the first time the use of Aphanothece sp. crude extract (ACE) as biostimulant to enhance plant antioxidant system and accumulation of metabolites against heavy metal stress. It may be a good strategy to restore contaminated soil without translate the H.M to the comestible part of plants for a healthy food^10,17,21–23^. In the present study, we investigated the effects of ACE as biostimulant on physiologic, biochemical, and metabolic responses of tomato plants to Pb and Cd stress.

## Results

In preliminary experiment studies, different concentrations of PbNO_3_ and Cd (NO_3_)_2_4H_2_O (0.1, 0.2, 0.4, 0.8, 1, 1.5 and 2 mM) were set to investigate the changes of morphological symptoms in tomato plant. Interestingly, no remarkable morphologic differences were found among individuals when exposed to low dose of heavy metals treatment (0–1 mM) for PbNO_3_ and Cd (NO_3_)_2_4H_2_O, while the plants were seriously hampered and grew abnormally when exposed to 2 mM PbNO_3_ and Cd (NO_3_)_2_4H_2_O (see supplementary Fig. S1). Therefore, we selected the 2 mM PbNO_3_ and Cd (NO_3_)_2_4H_2_O to study the effect of microalgae extracts on physiological, biochemical and metabolomic response of tomato plant under H.M stress. Furthermore, we testified three concentrations of crude extract (0.1%, 0.5% and 1%)of a cyanobacteria *Aphanothece* sp. and seven microalgae strains were *Chlorella vulgaris*, *Chlorella pyrenoidosa*, *Chlorella ellipsoidae*, *Chlorella sorokiniana*, *Scenedesmus obliquus*, *Scenedesmus dimorphus* and *Chlamydomonas reinhardtii* for their effect biostimulant against Pb and Cd stress*.* Thus, 1% of microalgae crude extract (MCE) supplementation to plant under 2 mM Pb and Cd stress showed a significant recovery of tomato root and shoot weight after their significant decrease under 2 mM Pb and Cd (see Supplementary Fig. S2). Indeed, we selected to treat in this paper the effect of *Aphanothece* crude extract (ACE), which composition in Table S3 as a cyanobacteria on physiological, biochemical, and metabolic responses under 2 mM Pb and Cd. Moreover, we choose to treat the effect of other microalgae separately in other study.

### Effect of microalgae ACE extract on tomato growth under Pb and Cd stress

**Figure 1:**
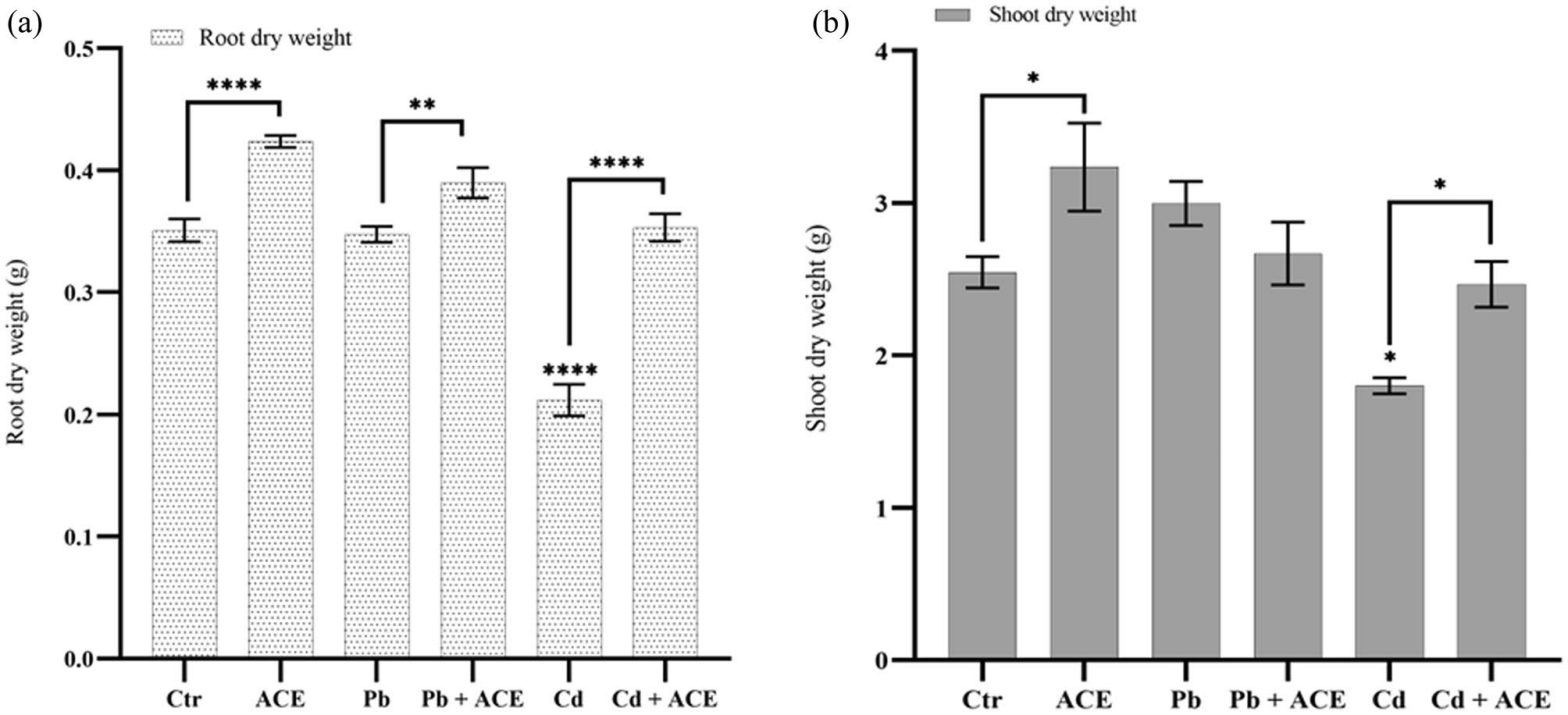
Effect of ACE on tomato root (**a**) and shoot (**b**) dry matter under Pb and Cd stress (Figure generated by GraphPad prism 9 software, while “*” indicates statistical significance (*P* < 0.05); “**” indicates statistical significance (*P* < 0.01); “****” indicates statistical significance (*P* = 0).

The effect of ACE on tomato plant growth under heavy metals stress was investigated in the present study, ACE application at sowing was not able to improve seed germination (data not provided here). While plants treated at four leaves stage has a remarkable difference between treatments. Thus, we used to apply our ACE on vegetative stage. In results, tomato plants exposed to 2 mM Pb and Cd stress showed no significant difference in tomato dry weights even for roots or shoots under 2 mM Pb. However, a significant reduction was noted in the dry weight of shoots and roots under 2 mM Cd by 29.25% and 39.48%, respectively compared to control (Fig. 1). Furthermore, the treatment of tomato under 2 mM Cd by ACE showed a significant recovery of dry weights shoot and root by 26.11% and 40.35%, respectively compared to plants under 2 mM Cd.

### Effect of ACE on photosynthetic pigments under Pb and Cd stress

**Figure 2:**
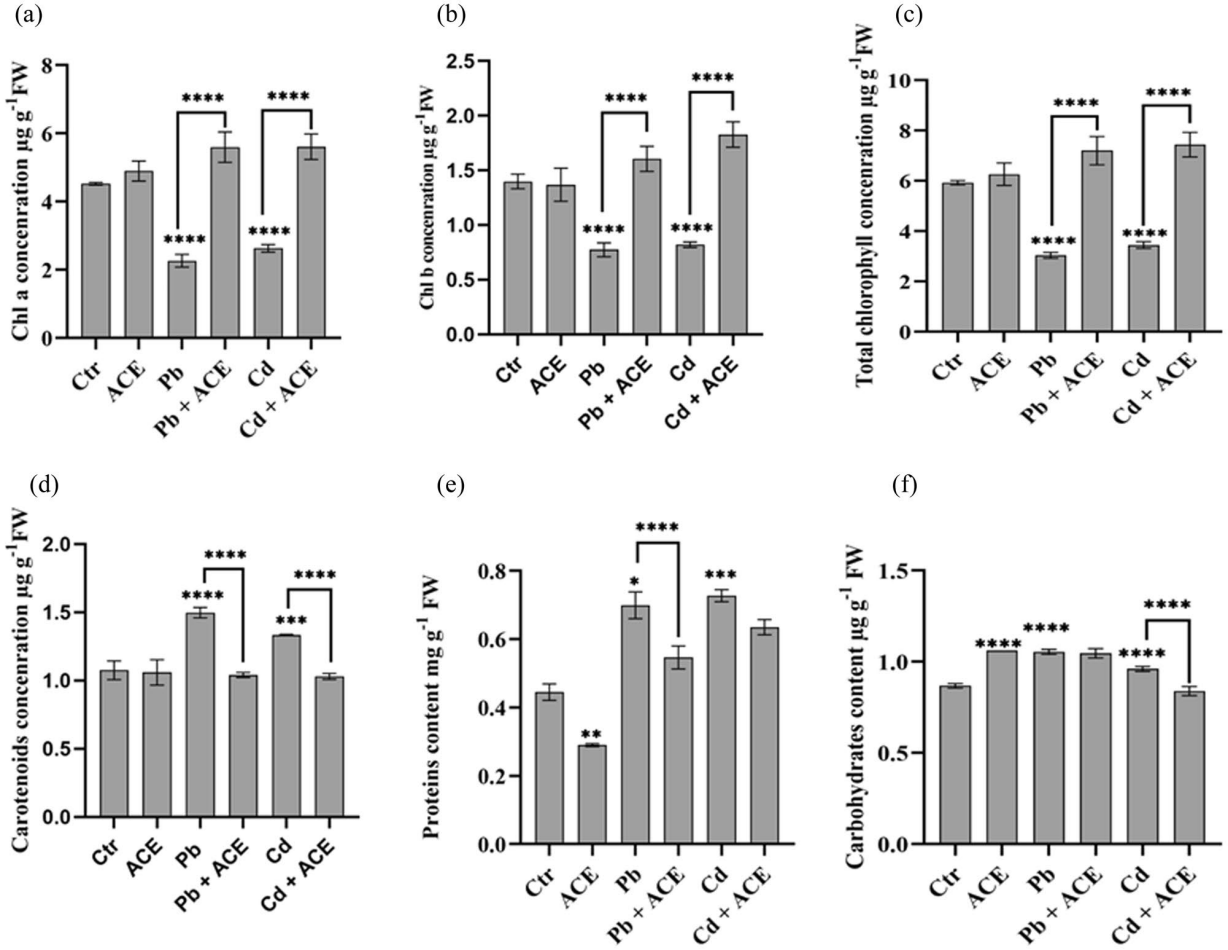
Effect of ACE on tomato leaves photosynthetic pigments (**a**) *Chl* a, (**b**) *Chl* b, (**c**) Total *Chl* and (**d**) Carotenoids and soluble proteins (**e**) and sugar (**f**). [Figure generated by GraphPad prism 9 software, while “*” indicates statistical significance (*P* < 0.05); “**” indicates statistical significance (*P* < 0.01); “***” indicates statistical significance (*P* < 0.001); *P* “****” indicates statistical significance (*P* = 0)].

The results presented in Fig. 2a–d demonstrate the effect of Pb and Cd stress alone and in combination with ACE on *Chl* a, *Chl* b, total *Chl* and carotenoids in the leaves of tomato plants. 2 mM Pb and Cd decreased significantly *Chl* a (49.97% and 41.92%, respectively), *Chl* b (44.73% and 41.37%), total *Chl* (48.73% and 41.79%) and significantly increased carotenoids (39.26% and 24.15%), respectively in tomato leaves. However, ACE supplementation normalized the content of chlorophyll pigments increasing significantly *Chl* a (23.70% and 24.15%,) *Chl* b (14.70% and 30.74%), total *Chl* (21.57 and 25.62%) and significantly decreased carotenoids content (3.15% and 4.18%).

### Effect of ACE on plant antioxidant system against Pb and Cd stress

**Figure 3:**
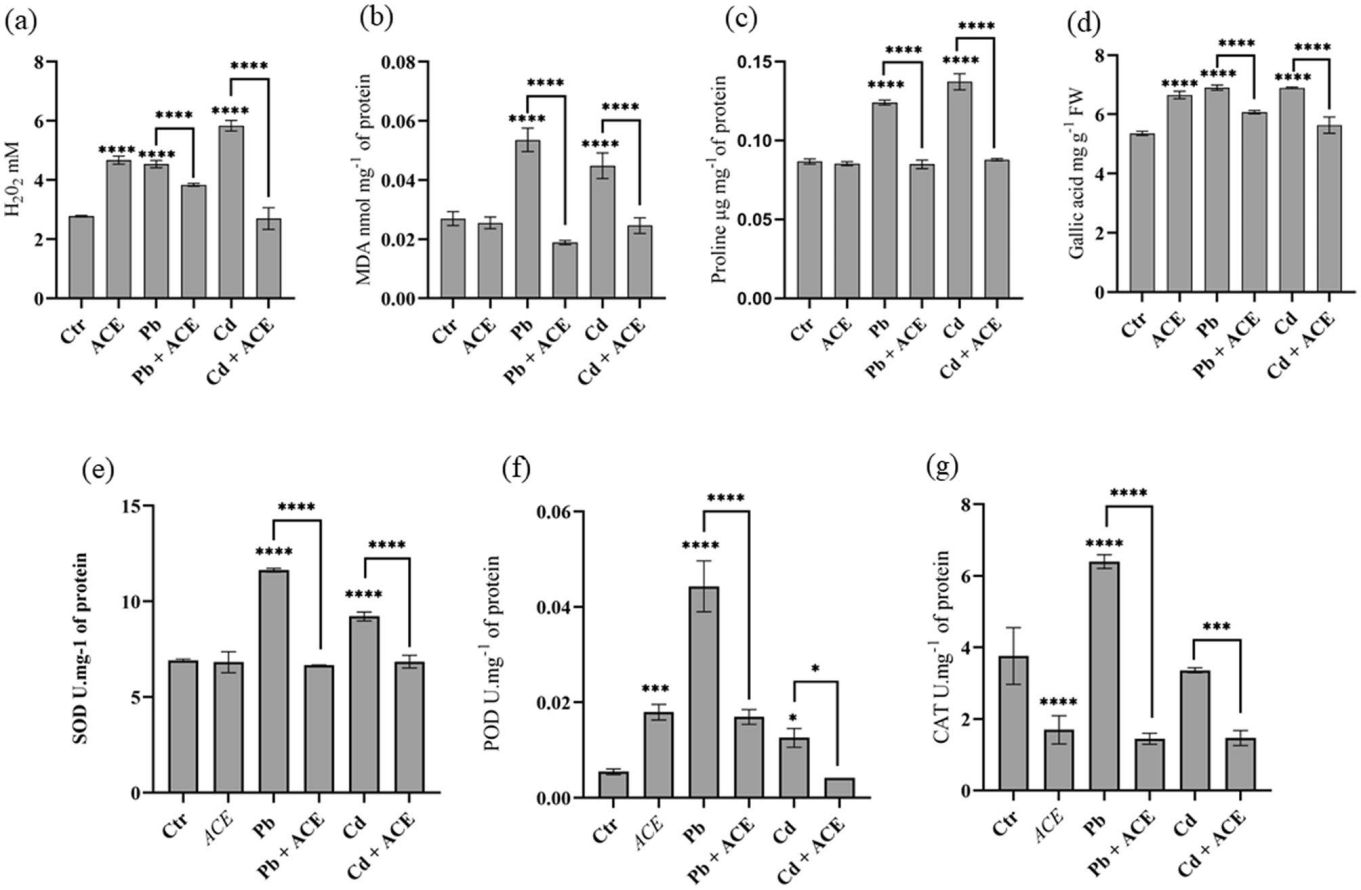
Effect of ACE on antioxidative system of tomato against Pb and Cd stress (**a**) H_2_O_2_, (**b**) MDA, (**c**) Proline, (**d**) Polyphenols, (**e**) SOD, (**f**) POD, (**g**) CAT. [Figure generated by GraphPad prism 9 software, while, “*” indicates statistical significance (*P* < 0.05); “**” indicates statistical significance (*P* < 0.01); “***” indicates statistical significance (*P* < 0.001); “****” indicates statistical significance (*P* = 0)].

In the present study, we investigated the effect of ACE on plant antioxidative system under 2 mM Pb and Cd. Tomato leaves showed significant elevation of H_2_O_2_ level with 63.45% and 110.15% under 2 mM Pb and Cd stress, respectively. Moreover, 2 mM Pb and Cd showed an increase of Malondialdehyde (MDA) level with 98.67% and 66.24%, respectively (Fig. 3a,b). Moreover, 1% ACE supplementation reduced significantly H_2_O_2_ and MDA level by 15.41% and 64.79% for Pb and 56.34% and 45.11% for Cd, respectively. Furthermore, the exposure to 2 mM Pb and Cd showed an accumulation of proline and polyphenols by 43.01% and 8.20% under 2 mM Pb, respectively and 58.21% and 9.26% under 2 mM Cd, respectively (Fig. 3c,d). In addition, ACE supplementation showed reduction in proline and polyphenols accumulation under stress by 31.46% and 7.56% under Pb + ACE in comparison to 2 mM Pb. For Cd, ACE application showed reduction of 35.98% and 15.16% in proline and polyphenols, respectively. 2 mM Pb and Cd exhibited significant increase of soluble sugar and proteins content in tomato leaves (Fig. 2e,f). However, ACE treatment normalized proteins and carbohydrates content in tomato leaves to meet control content. In parallel, we noted a significant increase of scavenging enzymes Superoxide dismutase (SOD) (68.23%; 33.28%), Peroxydase (POD) (759.25%; 150.63%), and Catalase (CAT) (93.14; 1.34%) under 2 mM Pb and Cd, respectively except CAT under Cd (Fig. 3e–g). Furthermore, ACE supplementation showed neutralization of all enzymes after their elevation under H.M stress (Fig. 3e–g). The lowest reduction recorded in SOD under 2 mM Cd + ACE with 25.65%.

### Effect of MCE on tomato plant metabolomic under H.M stress

**Figure 4:**
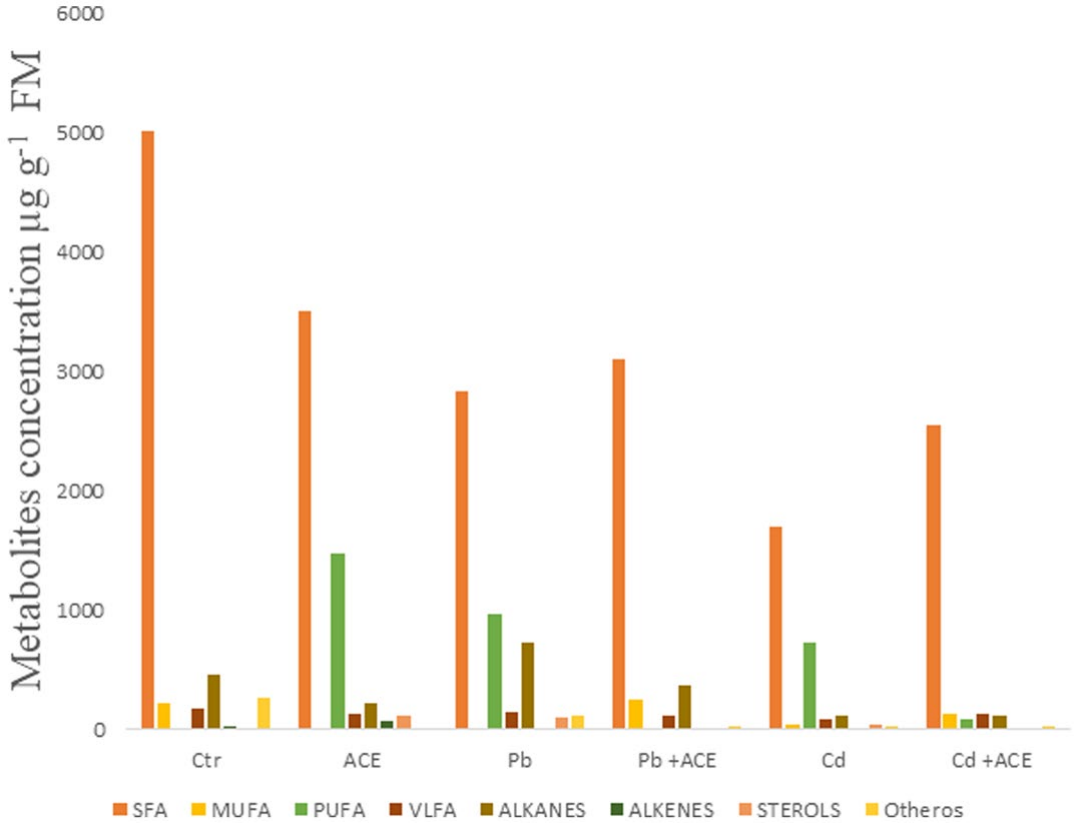
Effect of ACE on metabolites changes under Pb and Cd stress.

Metal stress application showed that 2 mM Pb and Cd reduced Saturated Fatty Acid (SFA) by 43.50%, and 66.16% and Monounsaturated Fatty Acid (MUFA) with 100% and 454.01%, respectively, while increased PUFA, alkanes and sterols compared to control (Fig. 4; Tables 1 and 2). However, ACE supplementation under Pb and Cd showed increase of SFA and MUFA, while decrease PUFA, alkanes and sterols (Fig. 4; Tables 1 and 2). Moreover, ACE application alone showed increase of PUFA, alkanes and sterols.

**Table 1:** Effect of ACE on FAME changes under Pb and Cd stress.

Metabolites (µg g^−1^)	Ctr	ACE	Pb	Pb + ACE	Cd	Cd + ACE
C8:0	44.835	–	–	–	–	–
C9:0	–	2.973	–	–	–	–
C14:0	20.636	16.000	14.130	15.913	10.030	13.464
C16:0	1655.103	1388.564	1096.607	1199.354	673.447	977.848
C17:0	–	–	–	14.936	–	–
C18:0	672.034	287.399	233.162	272.759	127.847	209.945
C20:0	140.367	67.065	75.677	50.484	39.747	74.184
SFA	5000.481	3505.033	2825.025	3090.983	1692.114	2537.420
C18:1	201.017	–	–	243.053	39.205	119.911
C20:1	16.189	–	–	–	–	
MUFA	217.207	–	–	243.053	39.205	119.911
C18:2	–	429.794	312.135	–	201.143	85.442
C16:3	–	37.065	–	–	–	–
C18:3	–	1003.487	652.646	–	514.508	–
PUFA	–	1470.346	964.782	–	715.651	85.442
C22:0	91.058	28.163	28.493	41.981	14.319	25.948
C24:0	49.653	80.856	80.211	69.472	43.953	44.673
C25:0	–	–	10.018	–	5.325	25.924
C26:0	21.405	16.171	15.496	–	10.780	23.001
VLCFA	162.117	125.191	134.219	111.453	74.379	119.547

**Table 2:** Effect of ACE on alkanes, alkenes, and Sterols changes under Pb and Cd stress.

Metabolites (µg g^−1^)	Ctr	ACE	Pb	Pb + ACE	Cd	Cd + ACE
Heptadecane	–	–	–	200,463	–	–
Octadecane	125,791	17,330	107,743	15,688	7137	85,464
Tetracosane	165,065	–	210,364	–	52,562	–
Penntacosane	–	–	59,667	–	–	–
Hexacosane	–	–	51,342	–	–	–
Heptacosane	69,850	–	51,342	–	–	–
Octacosane	27,898	–	55,779	–	–	–
Heneicosane	60,722	–	28,410	–	14,819	–
Eicosane	–	144,670	97,450	–	–	–
Nonacosane	–	–	–	29,086	–	–
Triacontane		53,221	59,152	120,791	41,279	25,305
Alkanes	449,325	215,222	721,249	366,027	115,797	110,770
1-Octadecene	10,771	–	–	–	–	–
Cholesta-6,22,24-triene	–	42,169	–	–	–	–
Cholest-5-ene, 3-methoxy-(3.beta)	–	28,962	–	–	–	–
Alkenes	10,771	71,131	–	–	–	–
Stigmasta-3,5-diene	–	92,666	25,241	–	–	–
y-sitosterol		12,230	–		8068	–
Stigmastan-6,22-dien, 3,5-dedihydro-	–	–	26,061	–	12,663	–
Stigmastan-3,5,22-trien	–	–		–	12,669	–
3,7,11,15-tetramethyl-2-Hexadecen-1-ol	–	–	40,493	–	–	–
Sterols	–	104,896	91,795	0.000	33,400	0.000
Other metabolites						
Furanone, 5 dodecyldihydro	148,663	–	–	–	–	–
Aalpha.-Tocopherol	40,581			19,575	8765	24,727
Alpha.-Tocospiro A	–	–	12,956	–	–	–
Tris(2,4-di-tert-butylphenyl) phosphate	67,859	–	33,285	–	13,968	–
1-(4-Fluorophenyl)-2-[(4-hydroxy-6-methylpyrimidin-2-yl)thio]ethan-1			65,229	–	–	–

### Effect of ACE on heavy metals accumulation and distribution in tomato plant

**Figure 5:**
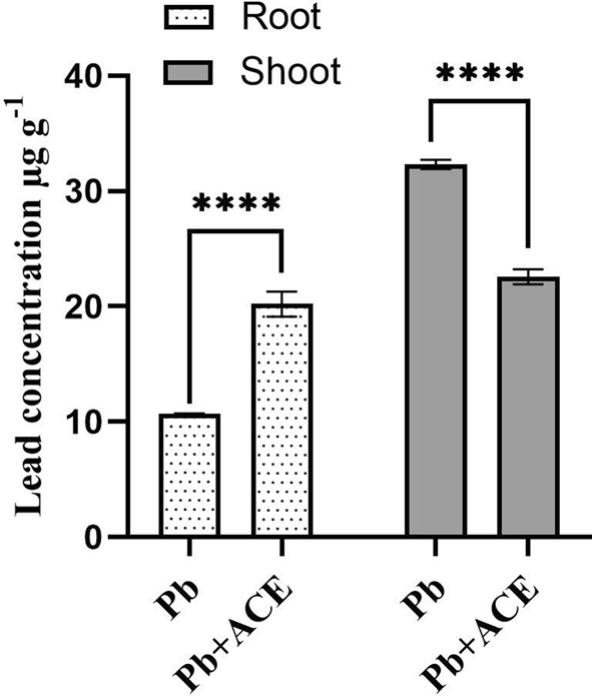
Effect of ACE on Pb and Cd uptake and translocation in tomato plant. [Figure generated by GraphPad prism 9 software, while, “****” indicates statistical significance (*P* = 0)].

ACE supplementation under Pb significantly enhanced the accumulation of Pb in root by 88.78% compared to 2 mM Pb. In shoot, ACE significantly reduced the accumulation of Pb by 30.18% compared to 2 mM Pb. Furthermore, ACE significantly reduced Cd concentration in root and shoot by 75.77% and 44.69% compared to 2 mM Cd (Fig. 5). Our results showed translocation inhibition from root to shoot, which tomato plants exposed to 2 mM Pb showed a Translocation Factor (TF) exceed 1, which was 3.01 ± 0.037. Moreover, ACE application to plant stressed by 2 mM Pb showed reduction in TF, which was 1.11 ± 0.028. For Cd, ACE showed in inhibition of Cd uptake and accumulation and both Bioconcentration Factor (BCF) and TF doesn’t exceed 1 (Table 3).

**Table 3:** Bioaccumulation and translocation factors of H.M.

	BCF	TF
Pb	0.008 ± 0.00	3.018 ± 0.037
Pb + ACE	0.008 ± 0.00	1.117 ± 0.028
Cd	0.006 ± 0.0003	0.270 ± 0.035
Cd + ACE	0.0018 ± 0.00	0.615 ± 0.037

### Effect of ACE on nutrients uptake and distribution under Pb and Cd stress

**Figure 6:**
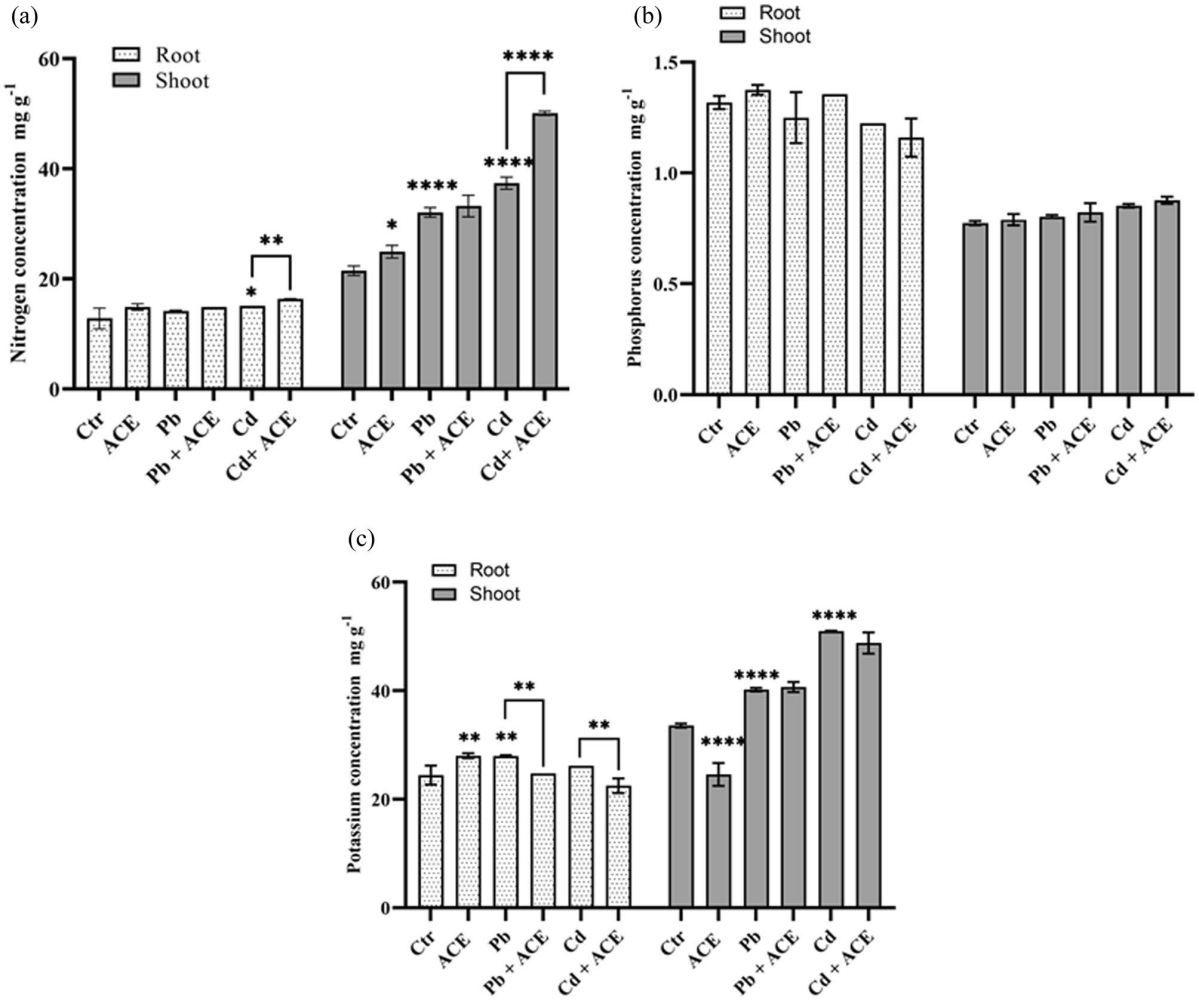
Effect of ACE on macro element N (**a**), P (**b**), K (**c**) uptake and translocation under Pb and Cd stress. (Figure generated by GraphPad prism 9 software, while “*” indicates statistical significance (*P* < 0.05); “**” indicates statistical significance (*P* < 0.01); “***” indicates statistical significance (*P* < 0.001); “****” indicates statistical significance (*P* = 0).

**Figure 7:**
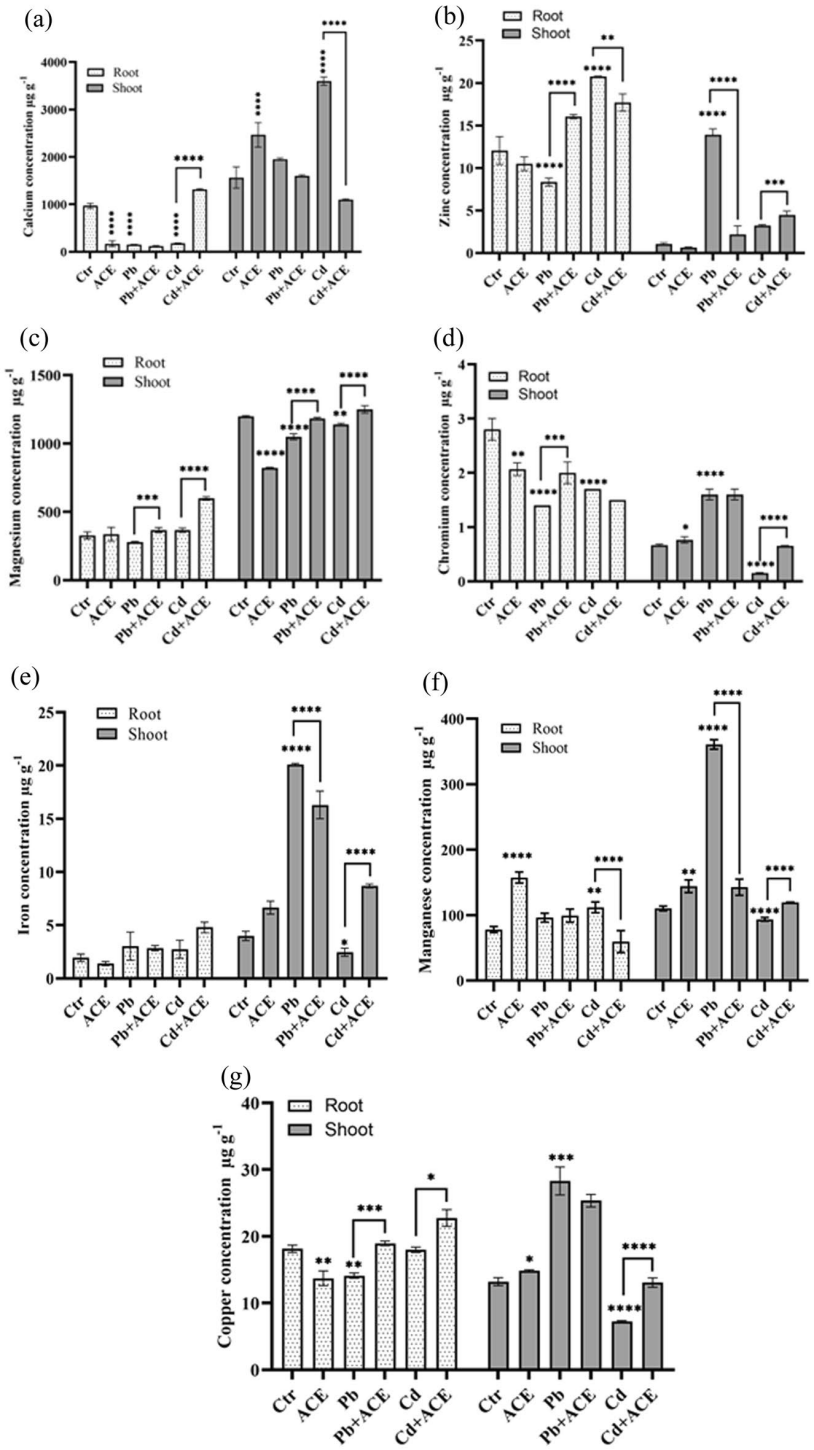
Effect of ACE on minerals element (**a**) Ca, (**b**) Zn, (**c**) Mg, (**d**) Cr, (**e**) Fe, (**f**) Mn, (**g**) Cu uptake and translocation under Pb and Cd. [Figure generated by GraphPad prism 9 software, while, “*” indicates statistical significance (*P* < 0.05); “**” indicates statistical significance (*P* < 0.01); “***” indicates statistical significance (*P* < 0.001); “****” indicates statistical significance (*P* = 0)].

In the present study we investigate the effect of ACE on nutrients uptake and redistribution under Pb and Cd stress (Figs. 6 and 7). ACE supplementation under 2 mM Cd enhanced the accumulation of Nitrogen (N) in root with 27.60% compared to control. Furthermore, ACE treatment enhanced N concentration in shoot to 54.64% and 133.01% under Pb + ACE and Cd + ACE, respectively. Phosphorus concentration showed no significant difference in root and shoot under metal stress and even after ACE supplementation. For potassium (K), the treatment by ACE significantly reduced K concentration by 11.38% and 14.19% under Pb + ACE and Cd + ACE, respectively compared to Pb and Cd. Moreover, ACE exhibited a significant increase of K in shoot by 21.19% and 45.28% under Pb and Cd, respectively compared to control. The effect ACE supplementation under Pb and Cd on other nutrients concentration was showed in Fig. 7. 2 mM Pb significantly reduced the concentration of Ca, Zn, Mg, Cu and Cr in root by 84.10%, 30.70%, 14.88%, 22.31% and 50%, respectively compared to control. ACE supplementation significantly increased the concentration of Zn (92.31%), Mg (31.78%), Cu (34.39%) and Cr (42.85%) compared to 2 mM Pb. 2 mM Cd significantly reduced the concentration of Ca (81.76%), Cu (0.82%) and Cr (39.28%) in root compared to control. Furthermore, ACE supplementation significantly enhanced concentration of Ca and Cu by 644.29% and 26.39%, respectively compared to 2 mM Cd. In shoot 2 mM Cd significantly reduce Mn, Mg, Cu and Cr concentrations by 15.10%, 4.91%, 44.88% and 77.08%, respectively compared to control. The concentration of these elements was increased significantly after treatment with ACE with Mn (27.91), Mg (9.67%), Cu (79.63%) and Cr (327.43%) compared to 2 mM Cd. 2 mM Pb significantly reduced Mg concentration by 12.49% compared to control, while enhanced after treatment by ACE with 12.71% compared to 2 mM Pb.

## Discussion

In the present study, the effect of ACE on physiological, biochemical, and metabolic responses under heavy metals stress has been investigated.

ACE application showed a significant recovery of shoot and root dry weights by 26.11% and 40.35%, respectively after reduction under 2 mM Cd by 29.25% and 39.48%, respectively compared to control. These results are in agreements with the finding of Abd El-All et al.^23^ showed that the treatment by seaweed extract (SWE) had a positive role in recovery to deleterious effect of heavy metals on tomato plant growth in comparison with control. respectively. In the light of this point, microalgae based biostimulant showed positive effect on plant growth and yield by reducing stress and restoring previous damages^24^. The biostimulant effect of microalgae extract was reported in numerous studies. Li et al.^11^, showed that 1% of microalgae extract enhanced significantly bean seedling growth. Tomato plants treatment by microalgae and cyanobacteria crude extract (CBEs) showed significant growth enhancement, particularly *Aphanothece* sp. which improved significantly root and shoot DW (34.81% and 58.69%), respectively^20^. The stimulation effect of microalgae extract may be due to the presence of plant growth promoting substances such as: macro-and micro nutrients, amino acids, fatty acids, polysaccharides, phytohormones etc., which can affect cellular physiology of plants enhancing plant growth and productivity^11,24–26^.

The result presented in Fig. 2a–d demonstrates the effect of Pb and Cd stress alone and in combination with ACE on *Chl* a, *Chl* b, total *Chl* and carotenoids in the leaves of tomato plants. ACE supplementation normalized the content of chlorophyll pigments increasing significantly *Chl* a (23.70% and 24.15%) *Chl* b (14.70% and 30.74%), total *Chl* (21.57 and 25.62%) after their decrease under 2 mM Pb and Cd, respectively. Moreover, ACE application significantly decreased carotenoids content (3.15% and 4.18%) after their increase under 2 mM Pb and Cd. In accordance, foliar application of seaweed extract resulted in a significant increase and recovery in chlorophyll content and carotenoids after harmful effect of H.M^23^. Furthermore, microalgae biomass used as fertilizer showed a positive effect in photosynthetic pigments contents^27^.

In the present study, 1% ACE supplementation reduced significantly H_2_O_2_ and MDA level by 15.41% and 64.79% for Pb and 56.34% and 45.11% for Cd, respectively after elevation by 63.45% and 98.67% for Pb and 110.15% and 66.24% for Cd, respectively (Fig. 3a,b). Moreover, ACE treatment normalized proteins and carbohydrates content in tomato leaves after their increase under Pb and Cd. Tomato plants treatment with K^+^ (5 mM) or Melatonin (50 µM) alone or combined showed significant reduction of H_2_O_2_ and MDA content under Cd stress with K (45%; 25%), Melatonin (36.36%; 17.85%) and K^+^ + Melatonin (49.09%; 42.85%)^28^. Moreover, microalgae polysaccharides showed improvement in H_2_O_2_ content, thus indicating a stimulatory effect on plant defense mechanism^19^. Microalgae extract showed reduction of H.M stress toxicity by lowering lipid peroxidation. In addition, ACE application alone showed increase of H_2_O_2_ level by 68.37% (Fig. 3a), which indicate that ACE stimulate plant oxidative system to reduce H_2_O_2_ and lipid peroxidation under H.M stress. In this regard, microalgae extract can modulate oxidative stress in plants^18,19^. Furthermore, microalgae contain antioxidant molecules such ascorbic acid, carotenoids, tocopherols, phycocyanin, and phenolic compounds. That plays an important role in redox homeostasis^29,30^.

Furthermore, ACE supplementation showed reduction in proline and polyphenols accumulation under stress by 31.46% and 7.56% under Pb + ACE in comparison to 2 mM Pb. For Cd, ACE application showed reduction of 35.98% and 15.16% in proline and polyphenols, respectively (Fig. 3c,d). In line of our results, selenium application as biostimulant at 1 µM under Cd stress reduced proline accumulation in tomato leaves^8^.

Scavenging enzymes are another barrier of antioxidative system played to reduce free radicals^31^. In the present study, we noted that ACE supplementation showed neutralization of scavenging enzymes (SOD, CAT and POD) after their elevation under H.M stress (Fig. 3e–g). In accordance, seaweed extract (SWE) application by foliar spray mode reduced significantly POD activity and in result recovered the deleterious effect of H.M^23^. Microalgae biostimulant contains bioactive molecules such as proline, betaines, phytohormones which can regulate plant redox homeostasis leading to stress resistance and tolerance^26^. Zinc oxide nanoparticles (ZnONPs) prepared from *Ulva lactuca* biomass at 25 mg^−1^ showed reduction of scavenging enzymes after elevation in *Leucaena leucocephala* leaves under Pb and Cd stress^22^. Moreover, tomato plants treatment by Ca (10 mmol L^−1^), salicylic acid (100 µmol L^−1^) and epi-brassinolide (1 mol L^−1^) in combination showed reduction of SOD, CAT and POD after their elevation under 5 mg L^−1^ of Cd^32^.

Our results showed that ACE can improve plant tolerance to H.M stress by stimulating plant oxidative system at early stage. ACE treatment alone showed elevation of H_2_O_2_ by 68.37% (Fig. 3a). However, ACE supplementation with Pb or Cd stress showed reduction of H_2_O_2_, MDA and scavenging enzymes activities after their elevation under Pb and Cd stress. Which indicate that plant early immunized for any stress by the first elevation of H_2_O_2_ from ACE. Furthermore, microalgae biostimulant act as elicitor contain bioactive molecules such as proline, betaines, phytohormones which can regulate plant redox homeostasis molecules leading to stress resistance and tolerance^33,34^.

ACE supplementation to tomato plants under Pb and Cd showed increase of SFA and MUFA, while decrease PUFA, alkanes and sterols after their augmentation under Pb and Cd stress (Fig. 4, Tables 1 and 2). Moreover, ACE application alone showed increase of PUFA, alkanes and sterols, which have a protective role against stress. PUFA, alkanes and sterols are the major metabolites that have a role in plant tolerance to abiotic and biotic stress^35^. PUFAs are the main compound of membrane, which keep plasma membrane permeability, integrity, and fluidity. They have also a role in ROS generation via activation of Nicotinamide adenine dinucleotide phosphate NADPH oxidase^36,37^. PUFA act also as the precursors of Lipoxygenases (LOX) pathway resulting in oxylipins biosynthesis after their peroxidation^38^. Oxylipin play the role of signaling and defense molecules^39^. In addition, the oxylipin induce expression of genes involved in the biosynthesis and accumulation of secondary metabolites^40,41^. Moreover, Alkanes are also a compound of membrane and wax cuticle, which play a vital role as a defence barrier against abiotic and biotic attacks^42,43^. Sterols have a role in membrane formation and preservation, against abiotic and biotic shocks^44^. Plants sterols can act as precursors of Brassinosteroids (BRs)^45^. Moreover, BRs improve plant tolerance to metal, thereby increasing crop yield and quality^46^. They can eliminate toxic metal via assisted phytoremediation system by plant growth regulators. BRs reduce H.M uptake via membrane permeability alteration, improve soluble proteins and increasing ATPase activity^47^. Moreover, they help in metal detoxification by enhancement of antioxidative system via scavenging enzymes and proline accumulation^46^.The accumulation of these metabolites can explain the role of ACE to alleviate H.M stress regarding to metabolites accumulated in comparison with plants under metal stress alone. In accordance, Rachidi et al.^19^ reported that microalgae polysaccharides showed redistribution of metabolites with improvement of lipids, alkanes, and sterols in tomato plants. Moreover, the treatment of tomato plant by liquid microalgae extract showed enhancement of SFA especially palmitic and stearic acid which are the first stage of de novo lipid synthesis^20^.

To investigate the effect of ACE on Pb and Cd accumulation, tomato plants exposed to 2 mM of Pb and Cd alone and combined with ACE. Thus, ACE supplementation under Pb significantly enhanced the accumulation of Pb in root by 88.78% compared to 2 mM Pb. Moreover, in shoot ACE significantly reduced the accumulation of Pb by 30.18% compared to 2 mM Pb. In accordance, spent mushroom compost (SMC) used as biostimulant of *Megathyrsus maximus* in contaminated soil improved significantly H.M uptake and remediation including Pb and Cd^48^. Furthermore, ACE significantly reduced Cd concentration in root and shoot by 75.77% and 44.69% compared to 2 mM Cd. In line of our results, the treatment by Ca (10 mmol L^−1^), salicylic acid (100 µmol L^−1^) and epi-brassinolide (1 mol L^−1^) in combination showed reduction of Cd concentration in root, stem and leaf^32^.

ACE application showed reduction of metal accumulation in root or/and shoot, which make ACE to be used for phytostabilisation strategy Indeed, the use of biostimulant directly in contaminated soil may have metal chelator effect, which enhance metal solubility and uptake by plants^48^. It may be due also to nutrients competition which ACE enhance nutrients uptake. In this regard, Gharaibeh et al.^49^ showed that zinc (Zn) combination with Cd may reduce metal concentration in different part of tomato plants. Moreover, potassium (K) supplementation at 310 ppm significantly reduced Cd translocation from root to shoot in tomato^50^. K supplementation confers plant exposed to Cd a positive response^32^. K can efficiently reduce Cd-toxicity and improve health of plant by enhancing photosynthesis activity and the biosynthesis of photosynthetic pigments^50^. Calcium (Ca) also reported as competitor ion of metals. The application of Ca at 2.5 mM in combination with Pb at 2.5 mM in soybean significantly reduced Pb accumulation in roots^51^.

Our results showed translocation inhibition from root to shoot, which tomato plants exposed to 2 mM Pb showed a TF exceed 1. Moreover, ACE application to plant stressed by 2 mM Pb showed reduction in TF. For Cd, ACE showed in inhibition of Cd uptake and accumulation and both BCF and TF doesn’t exceed 1 (Table 3). Even that the concentration found in tomato plants parts doesn’t exceed the limit imposed by the FAO/WHO for both Pb and Cd^52^. However, tomato showed the aptitude of metal uptake and translocation to the above part of plant, which represent a health risk to animal and humans. For this reason, an inhibition of metal uptake and translocation to the above part is necessary. Indeed, our study showed the potential of ACE as biostimulant of plant tolerance to heavy metals, which can minimize Pb and Cd uptake and translocation to the above part. In accordance, Gharaibeh et al.^49^ reported that fruits showed low Cd accumulation than shoot and root. Moreover, Eid et al.^53^ exhibited that TFs of H.M were < 1.0 in *L. esculentum*, while BCFs of H.M exceed 1 except for Al, Fe and Mn. Therefore, Metal accumulation may perturbate other nutrients uptake and distribution because of the competition in the transporters channel.

In this regard, we investigate the effect of ACE on nutrients uptake and redistribution under Pb and Cd stress (Figs. 6 and 7). ACE supplementation under 2 mM Pb and Cd increased NPK concentration in root and shoot compared to control. NPK are the most important nutrient for plant growth and development^54^. N is an important mineral element for plant productivity, which found mostly in nitrate, ammonium and organic molecules such as amino acids^55^. In the present study metal stress induced increase in N content in root and shoot even when added ACE. Moreover, our results showed increase of soluble proteins and enzymes activity which indicate the functional use of N assimilated by plant under each treatment. In accordance, Schreiber et al.^56^ showed the potential of *Chlorella vulgaris* as physiostimulators to enhance N and P accumulation in wheat plant. Generally, K deficiency decreased *Chl* a and b biosynthesis, which negatively affect plant growth and development^54^. In the present study, metals stress induced increase in K level in plant. However, we found a decrease in *Chl* a and b content in plant. This finding indicates that Pb and Cd affect the function of nutrients in photosynthetic pigments not the essential nutrients uptake. In accordance, Li et al.^57^ reported significant increase of K concentrations in welsh onion under 2.5 mg kg^−1^ Cd. Contrary, H.M stress showed a significant reduction of NPK concentration in soil irrigated with wastewater respectively^58^. Furthermore, the increase of some mineral concentrations under H.M stress might be attributed to their incorporation for H.M detoxification^57^. In conclusion, ACE may enhance nutrients uptake and translocation to minimize the uptake of Pb and Cd and detoxify these metals when they are present in plants parts, in turn enhance plant tolerance to metal stress.

The effect of metal and treatment by ACE on other nutrients concentration was shown in Fig. 7. ACE supplementation significantly increased the concentration of Zn (92.31%), Mg (31.78%), Cu (34.39%) and Cr (42.85%) after their reduction a under 2 mM Pb. Furthermore, ACE supplementation significantly enhanced concentration of Ca and Cu by 644.29% and 26.39%, respectively after their reduction under 2 mM Cd. In this regard, Asemoloye et al.^48^ reported that the use of SMC at 20% as biostimulant of *Megathyrsus maximus* enhanced nutrients status in contaminated soil by H.M. The use of *C. reinhardtii* and *C. sorokiniana* as biostimulant improved Mn and Cu, concentration in maize seedling^59,60^. Kusvuran^60^ showed that plant leaves treatment by *C. vulgaris* extract may affect positively plant nutrients content. The use of biostimulants may enhance nutrients uptake, which improve plant growth and productivity. Moreover, limiting the use of chemical fertilizers and protecting environment^21^. Numerous studies showed that biostimulant may protect plants from the excess and deficiency of nutrients^21,60,61^. Vernieri et al.^62^ reported that the application of biostimulant reduced the nitrate content in leaves. Moreover, microalgae may act as physioactivators, which can stimulate nitrate reductase and other enzymes incorporated in minerals absorption and transformation in plants^60^. Microalgae have bioactive molecules which has a major role in agriculture such improving nutrient uptake, physiological status, crop performance and abiotic stress tolerance^63,64^.

## Conclusion

Pb and Cd treatment reduced tomato growth and photosynthetic pigments through the generation of ROS and nutrients uptake disruption. However, ACE application showed corrective effect which increased tomato plant growth under Pb and Cd stress and enhance photosynthetic pigments. Tomato exposure to Pb and Cd showed increase in H_2_O_2_, which in results increase lipids peroxidation (MDA). ACE treatment showed reduction of H_2_O_2_, MDA and scavenging enzymes activities after their elevation under Pb and Cd stress. 2 mM Pb and Cd stress showed the reduction of SFA and MUFA, while increased PUFA, alkanes and sterols compared. However, ACE treatment under Pb and Cd stress showed increase of SFA and MUFA, while decrease PUFA, alkanes and sterols. Tomato plant showed significant accumulation of Pb and Cd in root. As well as a significant accumulation in shoot. Whereas ACE application favoured heavy metals accumulation in root and inhibited their translocation to shoot.Thus, ACE can be used as biostimulant of plant tolerance to heavy metals by minimizing H.M translocation to the aboveground part and can be used also in phytostabilisation strategy. Pb and Cd inhibited nutrients uptake and their distribution in plants. ACE application showed a positive effect in nutrients uptake and translocation under Pb and Cd stress. ACE alleviate metal stress by enhancing antioxidative system and nutrients status from the uptake to redistribution in different plant parts.

## Material and methods

### Microalga culture conditions and growth

The cyanobacteria *Aphanothece* sp. BEA O935B maintained at Algal Biotechnology Center at MAScIR (Moroccan Foundation for Advanced Science, Innovation and Research) collection was cultivated in BG11 medium^65^. The culture performed in triplicates using a 250 mL erlenmeyer flasks containing 150 mL of culture with a initial concentration of OD_680_ 0.1. The cultures were incubated at 25 ± 1 °C, 145 rpm of agitation (VWR Advanced Digital Shaker) and continuous illumination 100 µmol m^−2^ s^−1^ provided by white fluorescent lamps for 26 days. *Aphanothece* sp. growth was evaluated twice daily by measuring optical density (OD) at 680 nm using UV/VIS spectrophotometer brand Ultrospec 3100 pro.

### Aphanothece crude extract preparation

1.5 g dry *Aphanothece* sp. biomass was ground and suspended in 2% HCL. The mixture was heated for 2 h at 95 °C with stirring, alternated by sonication for 15 min each 30 min and autoclaved at 121 °C for 20 min. Aphanothececrude extract (ACE) was recovered as supernatant, which composition in Table S3 by centrifugation for 10 min at 2054 g and 4 °C and stored at − 20 °C (Centrifuge Heraeus Megafuge 40 R). The pH of supernatant was adjusted to 6.5 by KOH. ACE was tested on plants under 2 mM of Pb and Cd at three concentrations 0.1%, 0.5% and 1% for evaluation of their effect on plants cultivated in laboratory conditions at three replicates. Furthermore, the concentration 1% of ACE was selected based on their positive effect on agronomic parameters (root and shoot length and root and shoot weight) to investigates the effect of this extract on plant growth, chlorophyll content, polyphenols, biochemical changes, nutrient uptake and metabolomic profile of tomato plants under heavy metals stress.

### Partial characterisation of aphanothece crude extract

Proteins content in ACE was determined in 96 well-cell Plate Assay protocol according to Bradford^66^ method. Soluble sugar was quantified following phenol–sulfuric method^67^. The ACE was also characterised for NPK determination using Sakalar scan++ system at the Green Biotechnology Center, Mascir, Morocco. For heavy metals and mineral nutrient concentrations were estimated using inductively coupled plasma-optical emission spectroscopy (ICP-OES); (iCAP-7000 Duo, Thermo Fisher Scientific) at the Cereal and Legume Quality Laboratory, ICARDA, Morocco.

### Plant material and culture conditions

The seeds of the plant model *Solanum lycopersicum* L. var. JANA provided by BAYER Nunhems Netherlands BV were sown on 24 cell trays filled in commercial peat incubated in growth chamber with a day/night cycle of 16/8 h at 25 °C, 240 μmol photons m^−2^ s^−1^ and 69–72% relative humidity at green biotechnology laboratories in MAScIR. Uniform plants were selected and transported into six experimental groups with 10 biological replicates within each group following the standard procedures^68^. We declare that all vegetal materials was strictly used in the Green Biotechnology Laboratories certified ISO 9001 and OHSAS 18001 standards. Experimental research and field studies on plants (either cultivated or wild), including the collection of plant material, must comply with relevant institutional, national, and international guidelines and legislation. The experiment was organized as follow: (1) Control (without treatment), (2) Treated by ACE, (3) Treated with 2 mM Pb NO_3_, (4) Treated with 2 mM PbNO_3_ + ACE, (5) Treated with 2 mM Cd (NO_3_)_2_4H_2_O, 6. Treated with 2 mM Cd (NO_3_)_2_4H_2_O + ACE. The plants in first stage irrigated twice daily alternated with treatment and DW and daily in the last stage when plants were growth and require more water. The experiment is repeated twice time for confirmation. 40 days after transplantation, the plants were harvested and washed well a repeated time with DW, and the agronomic and physiologic parameters were evaluated.

### Biochemical parameters analysis

Photosynthetic pigments extracted and determined according to Xiong^69^ method. 0.1 g of fresh tomato leaves were grinded in liquid nitrogen using a mortar and pestle. The concentration of pigments was calculated according to the following formulae^70^:

1 $$ Chlorophyll a\; \left( { {\text{mg}}\,{\text{L}}^{ - 1} } \right) = 16.82 \cdot A665 - 9.28 \cdot A652 $$

2 $$ Chlorophyll b \;\left( { {\text{mg}}\,{\text{L}}^{ - 1} } \right) = 36.92 \cdot A652 - 16.54 \cdot A665 $$

3 $$ Carotenoids \;\left( {{\text{mg}}\,{\text{L}}^{ - 1} } \right) = \left( {1000 \cdot A470 - 1.91 \cdot Ca - 95.15 \cdot Cb} \right)/225 $$

Soluble proteins in tomato leaves was extracted according to Fleurence^71^ and determined following Bradford^66^ method by recording absorbance at 595 nm by Spectra Max Plus Molecular Devices spectrophotometer using BSA as standard. Total sugar was determined according to phenol–sulfuric method^67^. Total phenols were calorimetrically determined using Folin-Ciocalteu reagent as described by^72^.

### Determination of stress biomarkers H_2_O_2_, MDA and proline content 

The content of H_2_O_2_ was determined according to Velikova^73^ method with slight modifications. 0.1 g of fresh microalgal biomass was homogenized with 1 mL of 0.1% (w/v) trichloroacetic acid (TCA) on ice and centrifuged at 12,000×*g* for 15 min. 0.5 mL of the supernatant was mixed with 1 mL of 10 mM potassium phosphate buffer (pH 7) and 1 mL of 1 mM KI. H_2_O_2_ content was calculated using a standard curve after recorded absorbance at 390 nm. For proline content determination we followed Bates^74^ method. MDA content determined according to Heath and Packer^75^. 

### Scavenging enzymes assays

The enzyme extract was prepared by homogenizing 0.1 g of tomato leaves in 1 mL of 100 mM sodium phosphate buffer (pH 7.8), with 0.1 mM EDTA, 1% (w/v) polyvinyl pyrrolidone (PVP) and 0.5% (v/v) triton X-100. The crude protein concentration in supernatant was estimated by Bradford method using BSA as standard for protein quantification^66^. SOD activity was determined according to Beauchamp and Fridovich^76^ method. CAT activity was determined according to Aebi^77^ method. Then, POD activity was measured following guaiacol oxidation method^78^.

### GC–MS metabolomic analysis

To explore the tomato plants metabolomic responses under heavy metals (Pb and Cd) stress and ACE treatment, GC–MS method was performed following the described in^19^. Metabolomic analysis carry out by gas chromatography (GC) (Agilent 7890 A Series GC) coupled to mass spectrometry (MS) (Agilent 5975C) equipped with multimode injector and HP-5MS column with dimension of 30 m 250 mm 0.25 mm and electron impact ionization.

### Heavy metals and mineral nutrients analysis in tomato tissues

0.4 g of each ground sample in triplicates was placed in individual tubes and digested with 2.5 mL of H_2_SO_4_ at 100 °C for 2H, followed by adding 2 mL of 30% hydrogen peroxide (H_2_O_2_) carefully to each tube to complete digestion at 330 °C for 2H. Finally, after the sample solutions were cooled down, the volume was adjusted to 75 mL, and then filtered. The concentrations of NPK were determined by flow ionic analysis, Sakalar scan++ system at the Algal Biotechnology Center, Mascir, Morocco. 0.4 g of dry root and shoot were put it into digestion tube placed on aluminium heating block digester in triplicate. The digestion and mineralization of samples was done according to Gupta^79^ method. Heavy metals and mineral nutrient concentrations were estimated using inductively coupled plasma-optical emission spectroscopy (ICP-OES); (iCAP-7000 Duo, Thermo Fisher Scientific) at the Cereal and Legume Quality Laboratory, ICARDA, Morocco. Bioconcentration factor (BCF) and translocation factor (TF) were used to evaluate the potential of tomato to accumulate H.M within their root and the translocation to the above part. BCF and TF were determined following equation publishes in^80^:

4 $$ {\text{BCF}} = {\text{Heavy metal content}}\;\left( {\text{mg/kg}} \right)\;{\text{in the roots}}/{\text{Heavy metal content}}\;\left( {\text{mg/kg}} \right)\;{\text{in the soil}} $$

5 $$ {\text{TF}} = {\text{Heavy metal content}}\;\left( {\text{mg/kg}} \right)\;{\text{in a certain above - ground tissue}}/{\text{Heavy metal content}}\;\left( {\text{mg/kg}} \right)\;{\text{in the roots}}. $$

### Statistical analysis

Statistical analyses were performed using SPSS (IBM SPSS statistics 22). Descriptive statistics and significant differences of the mean values were determined using one-way ANOVA with a post-hoc Tukey’s and Duncan’s tests at a significant level of 0.05. While “*” indicates statistical significance (*P* < 0.05); “**” indicates statistical significance (*P* < 0.01); “***” indicates statistical significance (*P* < 0.001); “****” indicates statistical significance (*P* = 0). Graphs were generated by GraphPad prism 9 software (https://www.graphpad.com). All experiments in the work were performed in three replicates; the results were represented as arithmetic mean ± standard deviation.

## Supplementary Information

Supplementary Information.

## Data Availability

The data are available in the corresponding author for any request.
