# Supplementary material for: Aphanothece sp. as promising biostimulant to alleviate heavy metals stress in Solanum lycopersicum L. by enhancing physiological, biochemical, and metabolic responses
Source: Sci Rep. 2023 Apr 27;13:6875. doi: 10.1038/s41598-023-32870-4 (PMC10140289; doi:10.1038/s41598-023-32870-4)
Supplement: Supplementary file 1 — Supplementary Information. [file 41598_2023_32870_MOESM1_ESM.docx]

***Aphanothece* sp. as promising biostimulant to alleviate heavy metals stress in *Solanum lycopersicum* L. by enhancing physiological, biochemical, and metabolic responses.**

**Soufiane Fal^a,b*^, Abderrahim Aasfar^a^, Ali ouhssain^a^, Hasnae Choukri^c^, Abelaziz Smouni^b^ & Hicham EL Arroussi^a,d*^**

^a^ Green Biotechnology Laboratory, Moroccan Foundation for Advanced Science, Innovation and Research (MASCIR), Rabat Design Center Rue Mohamed Al Jazouli – Madinat Al Irfane, Rabat, Morocco.

^b^ Plant Physiology and Biotechnology Team, Center of Plant and Microbial Biotechnology, Biodiversity and Environment, Faculty of Sciences, Mohammed V University in Rabat, Rabat, Morocco.

^c^ International Center for Agricultural Research in the Dry Areas (ICARDA), Rabat, Morocco

^d^ Agrobiosciences Program, University Mohamed 6 Polytech

nic (UM6P), BenGuerir, Morocco.

^*^Corresponding authors: [soufiane_fal@um5.ac.ma](mailto:soufiane_fal@um5.ac.ma) and [h.elarroussi@mascir.ma](mailto:h.elarroussi@mascir.ma)


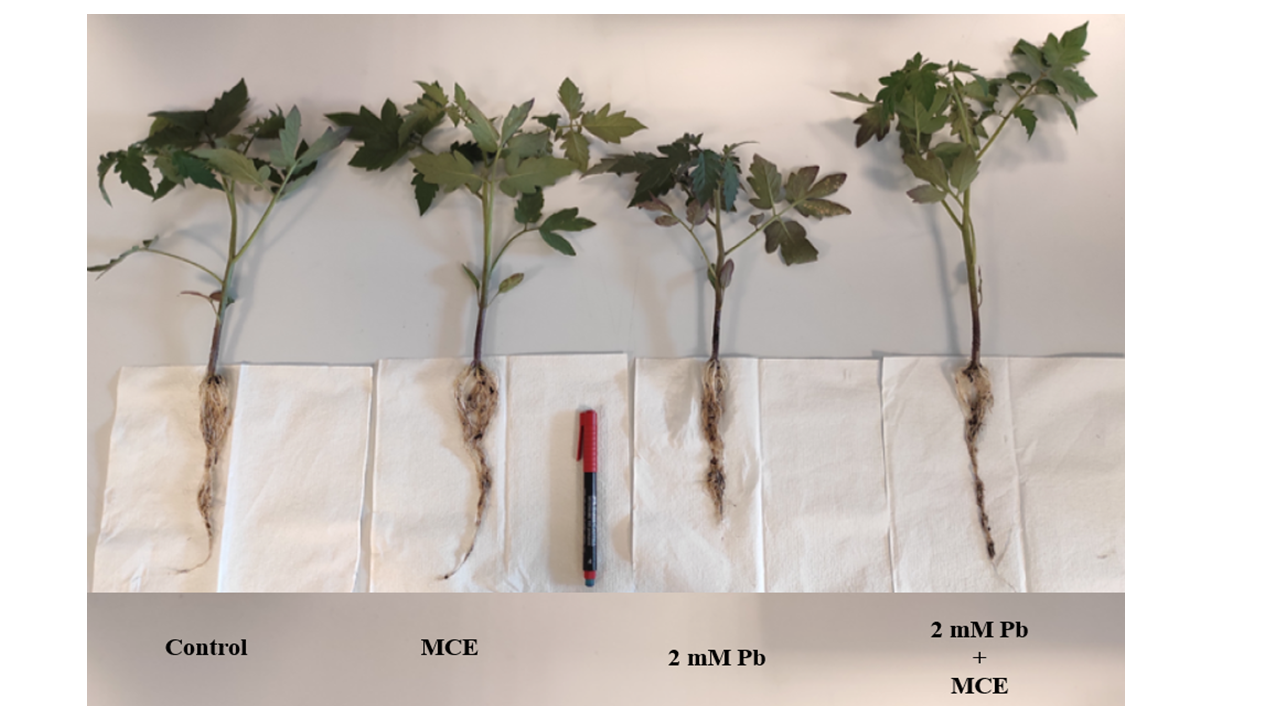


Fig. S1: Effect of MCE on agronomic parameters of tomato plants under Pb stress.


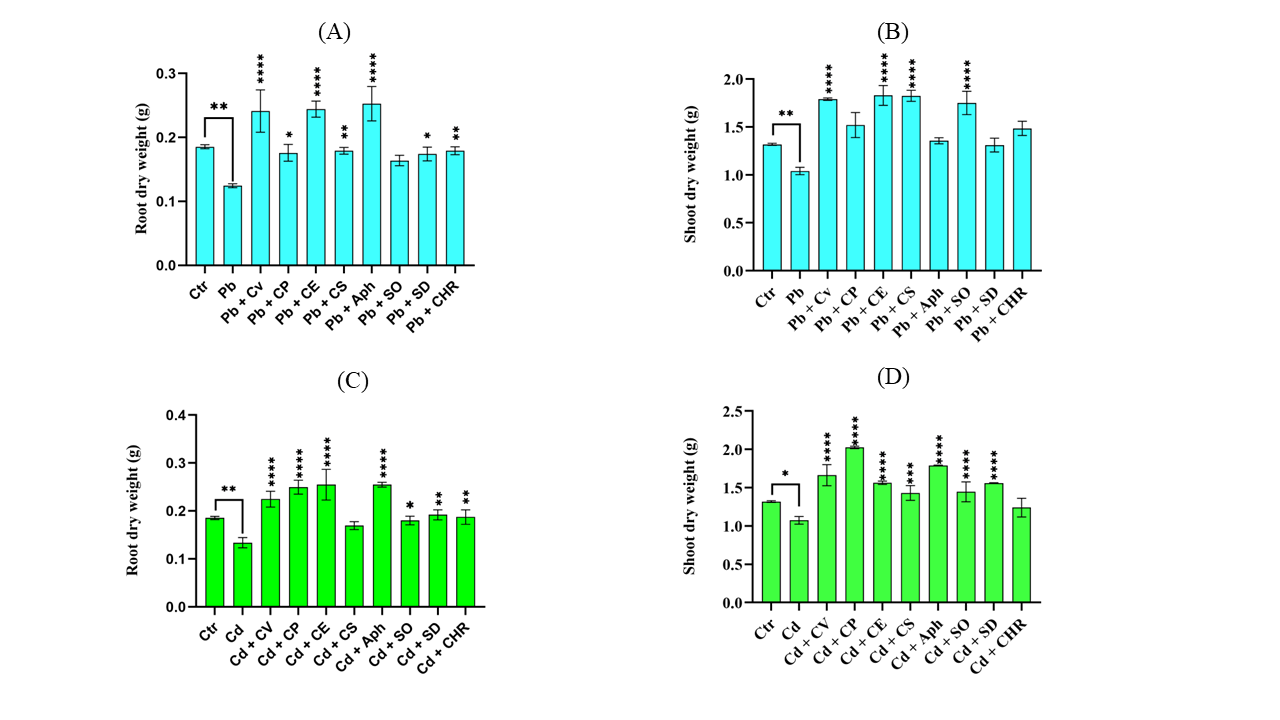


Fig. S2: Effect of MCE on tomato root (A, C) and shoot (B, D) dry matter under Pb and Cd stress. CV: *Chlorella vulgaris*, CP: *Chlorella pyrenoidosa*, CE: *Chlorella ellipsoidae*, CS: *Chlorella sorokiniana*, APH: *Aphanotheces* Sp., SO: *Scenedesmus obliquus*, SD: *Scenedesmus dimorphus* and CHR: *Chlamydomonas reinhardtii.*

Table S3: Composition of Aphanothece crude extract (ACE)

|  | *ACE* |
| --- | --- |
| Proteins | 0.054 |
| Soluble sugar | 0.049 |
| N (mg/ml) | 0.146 |
| P (mg/ml) | 0.226 |
| K (mg/ml) | 0.224 |
| Ca (mg/ml) | 0.164 |
| Zn (mg/ml) | 0.005 |
| Fe (mg/ml) | 0.021 |
| Mg (mg/ml) | 0.032 |
| Mn (mg/ml) | 0.137 |
| Cr (mg/ml) | 0.000 |
| Cu (mg/ml) | 0,002 |
| Pb (mg/ml) | 0,000 |
| Cd (mg/ml) | 0,000 |
